# Unmet diagnostic needs in cystic fibrosis infections and exacerbations: focus groups to inform target product profiles (TPPs)

**DOI:** 10.3310/nihropenres.13674.2

**Published:** 2025-12-02

**Authors:** Nicola Howe, Constance Takawira, Raasti Naseem, Kile Green

**Affiliations:** 1 NIHR Newcastle In Vitro Diagnostics Co-operative, William Leech Building, Newcastle upon Tyne, NE2 4HH, UK; 2 Medicines Discovery Catapult, Mereside, Alderley Park, England, SK10 4ZF, UK

**Keywords:** Cystic Fibrosis, Diagnostic Test, Respiratory Infection, Exacerbation, Focus Group, Target product Profile, AMR.

## Abstract

**Background:**

In addition to new antimicrobials for people with Cystic Fibrosis (pwCF), new diagnostics are needed to detect and diagnose infections, guide clinical care, and inform clinical decision making. To determine unmet diagnostic needs in pulmonary infection and exacerbation diagnostics in Cystic Fibrosis (CF), the required diagnostic test characteristics and priorities of different stakeholders involved in the care of pwCF were collected and analysed.

**Methods:**

Three focus groups (two clinical and one pwCF) were conducted and used to inform a wider project to deliver a suite of target product profiles (TPPs) for CF lung microbiological infection and/or exacerbation diagnostics. Thematic analysis was performed on the focus group data.

**Results:**

Participants described their experience of current practice and existing diagnostics for detection, diagnosis, and management of infection and exacerbations in CF in the UK National Health Service (NHS). Unmet needs included: monitoring modalities and testing for treatment efficacy; acquiring samples with good clinical utility; more acceptable sampling methods; and faster microbiology and culture-based testing.

Greater communication between the laboratory and clinical teams, and equity of care across UK CF centres was also highlighted. TPP characteristics of importance to pwCF and clinical representatives included ‘accuracy’, ‘time to results’, and ‘patient acceptability’. Both participants groups highlighted the need for suitable alternatives to sputum sampling and emphasised the need for novel biomarkers for the early detection and diagnosis of both infection and exacerbations. Amongst clinical representatives, test accuracy was generally valued over the time to results for a clinical test in a non-acute setting.

**Conclusions:**

Focus groups offered rich and detailed insights into the opinions of clinical staff and pwCF alike which informed further stakeholder engagement and shaped the content, scope and characteristics of TPPs. Early and rapid detection would have a positive impact on clinical care and inform earlier clinical decision making.

## Plain Language Summary

The overall health of people with cystic fibrosis is prone to rapid decline with the onset of each new or recurring lung infection they develop. Being able to diagnose a lung infection before symptoms persist, identify the strain and treat accurately and effectively with the right antibiotics provides the best opportunity to limit structural lung damage, irreversible decline, over medicalisation and speedier return to their normal routine, reducing impact on education, work or family commitments.

A focus group discussion with people living with CF and their carers heard why existing diagnostics do not meet their needs. Tests are unreliable, difficult to replicate, uncomfortably invasive, slow to give accurate results and leave clinicians uncertain about the appropriate course of treatment. This has a negative impact on the confidence of people with CF and increases anxiety. Focus groups with clinicians found the same concerns, identifying the need for new and improved methods of diagnosing lung infections to enable accurate clinical judgement when committing patients to a course of treatment with an ongoing consideration of limiting future treatment options through the development of antimicrobial resistance.

These findings were used as part of a wider project including questionnaire surveys and a symposium event to develop a suite of target product profiles (TPPs) (the ideal characteristics a test should have) for new diagnostic tests. The surveys and symposium included pwCF as well as healthcare professionals and developers of tests to ensure all views were heard.

## Introduction

Cystic fibrosis (CF) is an autosomal recessive inherited condition linked to mutation of the cystic fibrosis transmembrane conductance regulator (CFTR) gene. The effect of this defective gene protein is the build-up of mucus within the respiratory, reproductive and digestive system. In the lungs, this results in an increased predisposition towards recurrent infections and pulmonary exacerbations (worsening of symptoms) ( https://www.cdc.gov/cystic-fibrosis/about/index.html). Complications such as these can be difficult to diagnose, and frequently clinical manifestations are delayed such that lung function has already been compromised by the time of diagnosis. Over time, and with repeated infection and exacerbation, lung function degrades significantly. Despite treatment, some complex infections can become chronic illnesses that cannot be eradicated. Severe infections such as these can prompt an increased frequency of exacerbations, further impacting lung function and overall survival ( Stanford *et al.*, 2021). Timely diagnosis, treatment and management is key to maintaining adequate lung function for people with Cystic Fibrosis (pwCF).

Current clinical practice for diagnosing lung infections in pwCF is based on detection of pathogens in either sputum samples or cough/throat swabs using standard culture techniques. Samples are collected either during routine outpatient clinics (typically every 2–3 months in UK National Health Service centres) or during or following an episode of pulmonary exacerbation ( Smyth *et al*., 2014). However, a key limitation with current practice is the difficulty in obtaining suitable samples for testing. Some pwCF do not spontaneously produce sputum even when exacerbating, cough swabs are not recommended for diagnosing some types of infection, and whilst the alternative, bronchoalveolar lavage (BAL), is considered more reliable, it is also invasive and unsuitable for routine screening or surveillance ( Caverly, 2022). Cough and throat swabs, whilst less invasive, are more prone to test failure and are less effective in the detection of lower respiratory tract infections ( Caverly, 2022).

A significant proportion of UK pwCF are commencing highly effective modulator therapies (HEMT), including those early in childhood, resulting wide-scale changes to the presentation and frequency of complications in CF compared with those starting HEMT later in life who may already have established chronic lung disease ( Bell *et al*., 2020). As those on HEMT generally feel ‘healthier’ with the reduction in symptoms associated with CF, they are also less likely to produce sputum on demand, driving a need for the CF community to find suitable alternatives to standard sputum culture testing. 

Novel detection methods which reduce reliance on sputum collection do exist but are not yet widely available in clinical practice and the acceptance or tolerance of such diagnostic tests among pwCF is paramount to their successful use in both community and healthcare settings. Developers therefore need to engage with the CF community to develop a clear understanding of the needs of pwCF, as well as the clinical utility of a test. The CF population represents an accessible and well-defined group for product development due to the presence of specialist CF centres in the UK and other countries, as well as an active patient registry and clinical trial network ( Matthews *et al*., 2024).

Jointly managed by the CF Trust, the Medicines Discovery Catapult (MDC) and LifeArc, the CF Antimicrobial Resistance (AMR) Syndicate was established to accelerate the translation and adoption of new CF antimicrobials and diagnostics to the clinic through collaboration, bringing better treatment options to people with CF, faster. This project brought together the complementary expertise of the NIHR Newcastle *In Vitro* Diagnostics Co-operative (NIHR MIC) (now NIHR Health Technology Research Centre (NIHR HRC)), and the CF AMR Syndicate to deliver a suite of target product profiles (TPPs) for CF infection and exacerbation diagnostics.

This work builds on the previous scoping study conducted during the early phase of this collaborative project ( Naseem *et al*., 2024) to better understand the requirements for diagnosing infections in pwCF. Here we describe the qualitative outputs collated during expert and patient focus groups discussing the CF care pathway, positive and negative aspects of clinical care, unmet needs in CF, and the perspectives of pwCF and clinical teams on optimal and desired characteristics for diagnostic tests in CF.

The patient-centric methodology builds upon the experience and network of MDC and CF Trust in the development of TPPs to guide CF antimicrobial therapeutic development. These early stakeholder focus groups informed all subsequent diagnostic TPP development work, and laid the foundation for subsequent stakeholder elicitation regarding content and focus, culminating in a stakeholder symposium.

Table 1 contextualises the focus groups described in this manuscript in the wider collaborative TPP development project. Full details of the methodological approach taken and how focus group findings informed subsequent work on TPP development is available in our methodology publication ( Green *et al.*, 2025).

**Table 1.** Placing the focus groups in the context of the Target Product Profile development project. This article describes the activities and outcomes of the focus group portion of Phase 1 of the TPP development process (highlighted in blue). These focus groups aimed to define and prioritise the unmet diagnostic needs affecting healthcare professionals and people with lived experience of cystic fibrosis. The prioritised focus groups would then inform the TPPs to be developed in the later phases.

Study Phase:	Phase 1- Landscape Analysis	Phase 2- Target product profile (TPP) Drafting	Phase 3- Refinement and validation
Purpose:	*To identify and prioritise unmet needs*	*To address unmet diagnostic needs*	*To refine language, use- case, and determine fit for purpose*
Activities:	**Rapid literature reviews** (Guideline review and scoping review)	**Focused document review** (existing target product profiles, regulatory documents)	**Additional interviews** (industry and developers, people with CF and clinical teams, charity groups)
	**Focus groups** (lived experience, people with CF, NHS clinical groups)	**Expert elicitation** (focus groups, interviews)	**Two-round Delphi** (Non-tuberculous mycobacteria experts, industry, regulatory experts & people with CF)
	**Expert discussions** (Target product profile experts, methodologists)	**Evidence generation** (surveys and questionnaires, iterative feedback	**Virtual symposium** (breakout rooms, keynote speaker with CF)

## Methods

### Patient and Public Involvement

Following on from the research priorities set out by the James Lind Alliance ( https://www.jla.nihr.ac.uk/priority-setting-partnerships/cystic-fibrosis) who consulted with pwCF, family members and clinical care teams globally, the project team wanted to further understand the diagnostic unmet needs and priority areas for clinicians and pwCF, and investigate where, and what type of new diagnostics tests might have the most patient and UK National Health Service (NHS) benefits.

For the focus groups informed consent forms and participant information sheets were drafted and revised following consultation with a patient and public involvement and engagement (PPIE) manager and the NIHR Newcastle MIC insight public panel members. Insight panel members were also given an overview of the project and it’s aims and were invited to comment on the methodology and later the first draft of the TPP document, which was was subsequently revised based on their feedback.

This project also involved an expert advisory group comprised of people with lived experience of CF, clinicians, methodologists expert in TPP development, CF charity members and academics with an interest in CF and microbiology. The panel met formally approximately 7 times throughout the duration of the project, with supplementary ad-hoc meetings and online document review conducted as required. Output from work at each stage of the project was shared with the group including drafts of the TPP document, which was revised accordingly. The focus groups described here were also attended by a PPIE Coordinator from the CF Trust with lived experience of CF, who provided contributions and facilitation.

A virtual symposium held after the focus groups and not described here had a pwCF as a keynote speaker and pwCF in all breakout room and sessions.

### Participant selection

18 participants were recruited through existing networks; through the CF trust, or personal networks of the authors (convenience sampling) and invited to take part in the focus groups via email. Participants were based across different regions of the UK. Anonymised details of the focus group members with CF are outlined in Table 2 and details of clinical care team focus group members are given in Table 3 and Table 4.

**Table 2.** Participants with CF and their loved ones.

Identifier Code	Demographics
pwCF_1	Female, person with CF
pwCF_2	Female, person with CF
pwCF_3	Female, person with CF
pwCF_4	Male, person with CF
pwCF_5	Male, parent of young person with CF
pwCF_6	Male, person with CF

**Table 3.** Clinical focus group 1.

Identifier Code	Job title	Years of Experience
CFG1_1	CF Physician (adult)	20+ years of experience, 8 years as a specialist
CGF1_2	CF Physiotherapist (adult & paediatric)	20+ years of experience.
CGF1_3	CF Clinical Nurse Specialist (CNS) (adult)	5+ years of experience, 2 years as a CNS
CGF1_4	CF Physician (adult)	15+ years of experience
CFG1_5	Consultant Chronic Suppurative Lung Diseases (CSLD) pharmacist (paediatrics)	20+ years of experience
CFG1_6	Senior biomedical scientist	30+ years of experience

**Table 4.** Clinical focus group 2.

Identifier Code	Job title	Years of Experience
CFG2_1	CF Physician (adult)	10+ years of experience
CFG2_2	CF Physician (paediatrics)	25+ years of experience
CFG2_3	CF Nurse (paediatrics)	15+ years of experience
CFG2_4	CF Physiotherapist (adult)	15+ years of experience
CFG2_5	Lead CF Pharmacist (adult)	10+ years of experience
CFG2_6	Senior biomedical scientist	20+ years of experience

### Focus group format

Consent was taken verbally prior to the focus group commencing by one researcher, and witnessed and recorded by a second researcher. All participants consented to the recording of the interviews.

Focus groups were conducted using Microsoft Teams. For each focus group, in addition to the lead interviewer (KG) at least one additional researcher was present to act as facilitator and note taker. Six participants per focus group was selected to allow a range of views but allow time for participation by all present.

One 90-minute focus group was conducted with a total of six participants with lived experience of CF on 13 ^th^ July 2022.

Two 120-minute focus groups were conducted with six clinical participants in each on 20 ^th^ and 24 ^th^ October 2022.

A lay summary explaining the purpose and design of TPPs was produced and distributed prior to, and during, the pwCF focus group (extended data). The clinical participants were provided with an agenda and briefing document prior to the clinical focus groups (extended data). Separate focus group topic guides were designed for clinical staff and pwCF.

Topic guides ensured the focus groups addressed the main objectives including:

- Unmet needs in CF care.
- Perspectives from pwCF and clinical care teams on optimal and desired diagnostic test characteristics.
- The clinical care pathway during exacerbation and infection.
- Positive and negative aspects of clinical care of pwCF.

### Analysis

Audio recordings were auto-transcribed by the online web-service otter.ai ( https://otter.ai/). These auto-generated transcripts were cleaned by author KG and checked for accuracy by all authors. Any outstanding issues or clarifications were resolved with the focus group participants. Summary content and themes were extracted from the transcript to aid in early project decision making and to inform the subsequent analysis. 

A thematic analysis as described by Braun and Clarke ( Braun & Clarke, 2006) was performed on the transcripts and notes taken during the interviews. Transcripts and notes were coded by hand by author KG and annotated to identify themes, after which an electronic thematic summary document was produced with relevant data extracted under each key theme. Themes were discussed and agreed with authors CT and RN. Themes broadly reflected the key questions asked during the focus group plus additional topics important to participants. A thematic summary document was produced for the clinician focus groups and a separate document was produced for the pwCF focus group. Both were checked for accuracy and completeness and agreed by all researchers who facilitated the focus groups. Results are presented in a narrative synthesis.

Quotes from focus group participants have been anonymised and appear in bold italicised text below.

## Results

Key themes were determined as: Detection and diagnosis of infections and exacerbations in pwCF, Current diagnostic tests, interactions between pwCF and clinical care teams, Unmet needs in CF infection and exacerbation and stakeholder preferences in TPP characteristics. For the theme ‘current diagnostic tests’ we have included an author description in Table 5 of current testing methods identified by participants, after which follows the participants discussion of each testing method regarding their perceived use, accuracy, and acceptability.

**Table 5.** Contextual information on identified diagnostics.

Identified diagnostic	Diagnostic type	Population	Indication	Setting	Time to results
Cough swabs	Culture	Adults and children unable to expectorate sputum. Predominantly used in paediatrics.	First-line diagnostic (in those unable to expectorate sputum), routine, symptomatic and asymptomatic patients.	At-home, GP/primary care, community, secondary care.	Minimum ~3 days, (considered less sensitive than sputum for detection of different pathogens).
Sputum-based culture methods (sputum, induced sputum)	Culture	Adults and children with CF, predominantly adult populations	First-line diagnostic, routine, symptomatic and asymptomatic patients.	At-home, GP/primary care, community, secondary care.	Minimum ~3 days, up to 6–8 weeks, depending on infection and pathogen type.
Bronchoscopy and BAL	Imaging/ Culture	Symptomatic adults and children, where infection persists despite treatment, or where initial diagnostic tests have proved inconclusive.	Second-line diagnostic, non-routine, emergency, ad-hoc	Secondary care, specialist centres, emergency department	Typically 1–3 days for reporting; ~3 days, or up to 6–8 weeks, depending on infection and pathogen type
Spirometry	Surrogate biomarker	Adults and children with CF	First-line diagnostic, routine, monitoring, symptomatic and asymptomatic patients.	At-home, GP/primary care, community, secondary care.	Immediate
X-ray and CT scans	Imaging	Symptomatic adults and children with CF, where infection persists despite treatment, or where initial diagnostic tests have proved inconclusive.	Second-line diagnostic, non-routine, emergency, ad-hoc	Secondary care, specialist centres, emergency department	Typically 1–3 days for reporting.
Magnetic resonance imaging (MRI) scans	Imaging	Symptomatic adults and children with CF, where infection persists despite treatment, or where initial diagnostic tests have proved inconclusive.	Second-line diagnostic, non-routine, emergency, ad-hoc	Secondary care, specialist centres, emergency department	Typically 1–3 days for reporting.
Blood tests	Surrogate biomarker	Adults and children with CF	First-line diagnostic, routine, monitoring, symptomatic patients	GP/primary care, community, Secondary care, specialist centres, emergency department.	Typically 1–5 days for reporting.
Passive monitoring techniques	Surrogate biomarker	Adults and children with CF	Routine, ad-hoc, continuous monitoring, Symptomatic and asymptomatic patients.	At-home	Immediate, continuous

### Detection and diagnosis of infections and exacerbations in pwCF

***The impact of HEMT***

Elexacaftor/Tezacaftor/Ivacaftor (ETI) is licensed for use in pwCF with at least one F508del mutation in the CFTR gene. There remains a population of pwCF who are unable to benefit from HEMT like ETI. Disease causing CFTR gene mutations are responsible for the respiratory symptoms associated with CF, namely the production of thick, sticky mucus in the airways which is difficult to clear and leads to recurrent respiratory infections. ETI works to increase the activity of the CFTR proteins on the cell surface, leading to a reduction in pulmonary exacerbations and an improvement in quality of life During this elicitation exercise, both pwCF and the clinical care teams stated noticeable changes in the frequency of pulmonary exacerbations, as well as changes in the symptom associated with acute illness following the roll out of ETI.

***“After Kaftrio [ETI]… I have a normal person cough or cold, where if I have something on my chest, I go [cough], and it moves, and I can test it” – pwCF_1***

When considering detection and diagnosis of pulmonary exacerbations in the era of HEMT, many pwCF have experienced changes to their symptom profile. Sputum production has decreased for many following initiation of HEMT, resulting in fewer expectorated sputum samples. This in turn has led to a greater reliance on obtaining induced sputum and cough swabs for detecting and diagnosing infections

***“Since Kaftrio [ETI], we've been doing more induced sputum, more cough swabs, and even our induced sputum approach at the moment, isn't always productive.” – CFG2_2***
***“With the new modulators, a lot of people aren't actually producing any sputum anymore. So it's hard to get anything other than a cough swab” – CFG1_6***

***Symptoms of infection and exacerbation***

Participants in the pwCF and clinical team focus groups described subjective and objective signs and symptoms used to diagnose infection and exacerbation. Some of these were common, such as an increased cough, body pains, fever and inflammation, but others were person-specific, including post-nasal drip, migraines and dry, cracking skin on the lips and knuckles. With other reported symptoms including pleuritic pain, exhaustion, loss of taste (Ageusia) and loss of smell (Anosmia), diagnosis of infection based on signs and symptoms alone can be challenging. In addition, the importance of distinguishing a chronic infection from an acute exacerbation was flagged.

Other indicators of possible exacerbations are linked to monitoring tools. A decline in lung function noted by pwCF based on home spirometry can be an early indicator of a potential exacerbation, alongside changes to sputum colour, volume and consistency. Some participants in the pwCF group described noticing changes to their resting heart rate in the days leading up to the onset of symptoms. Continuous home monitoring of heart rate is part of ongoing CF trials such as Project Breathe ( https://www.lifearc.org/2023/nhs-trial-uses-artificial-intelligence-to-predict-severe-infections-and-transform-lives-of-people-living-with-chronic-lung-conditions/), although no data has been released on the correlation between resting heart rate and pulmonary exacerbations, it is used by pwCF and their clinical teams as an early indicator of acute illness. Participants in the pwCF focus group also reported having a ‘gut feeling’ of impending illness that would lead them to seek antibiotic treatment from their care team.

***“So [there are] objective markers - FEV1, saturations maybe, but then really a lot of it is subjective” – CFG1_4***
***“I always feel like when I've got something, there's just there's a gut instinct that says ‘This isn't normal’... that's what tells you what's wrong” – pwCF_3***

### Current diagnostic tests

There are a range of diagnostic tests currently in use in the UK NHS for diagnosing infection or exacerbation in pwCF. For example, sampling of airway secretions is considered ‘routine’ and will be performed during clinic visits in both symptomatic and asymptomatic cases. Others, including imaging techniques and more invasive procedures such as bronchoscopy, are performed ad-hoc, when additional information is required to aid decision making. Blood tests, i.e. whole blood counts, and spirometry, while not diagnostic tests for infection per se, are used as part of a series of tests which when viewed collectively assist in diagnosing acute illness alongside culturing techniques.

In this section the included tests were those elicited through the focus groups. Table 5 includes a description of each by the authors. Each identified diagnostic is considered in turn, highlighting features and characteristics of the tests and opinions from pwCF and clinicians regarding their perceived use, accuracy and acceptability.

***Cough swabs***

**Elicitation - Use:** Cough swabs were generally performed by a specialist nurse or physiotherapist, and were more commonly used in paediatrics, however since the roll out of HEMT, use of cough swabs in the adult population has risen as many are no longer able to expectorate sputum on demand. The samples would be sent via courier or internal post to a microbiology laboratory for analysis. Cough swabs are taken regularly both during routine clinic visits and during periods of infection.

***“So we get cough swab samples on our babies and young children regularly, every clinic visit, every six to eight weeks, more frequently in young babies soon after diagnosis.” - CFG2_2***

**Elicitation - Accuracy:** Participants in all focus groups believed cough swabs were not accurate or reliable and would not normally be used in isolation. One clinical representative (CFG1_2) stated that cough swabs had a good positive predictive value, but a poor negative predictive value, indicating that a positive result usually indicated an infection, but with a negative result, clinicians would not be confident enough to rule out an infection based on a cough swab alone.

***“From a physio perspective, cough swabs are next to useless, unless you have no other way of obtaining anything.” – CFG2_4***

The inaccuracy of cough swabs, particularly where there is a negative result, makes decision making around antibiotic usage more difficult. This impacts decisions such as whether or not to continue therapies for chronic infection, especially as some pwCF wish to discontinue their treatments on the basis of negative cough swabs which the clinical care team consider unreliable as a standalone result. Across the clinical care team, there was consensus around the worry of false negatives when it came to cough swabs

***“We do find it quite difficult at the moment when patients are saying they'd like to stop certain antibiotic treatments because they haven't grown Pseudomonas in the last year, but they've only got negative cough swabs as opposed to sputum samples” – CFG2_4***

**Elicitation - Acceptability:** In regard to methods of sampling, cough swabs were the least invasive method according to the focus group attendees, they were commonly used in the paediatric settings. They were considered the most tolerable test in adults and children.

***“From the outreach clinics, it's normally cough swabs, which aren't ideal… cough swabs and cough plates are inferior specimen types to sputum, but it's obviously easier to [collect and] send us those specimens.” – CFG2_6***
***“We use cough swabs a lot, on babies, it's an induced cough swab, so [we] will touch the back of the throat. And then in children that can cough, we will use that. It's very difficult to get sputum from them.” – CFG2_3***

**Elicitation - Other comments:** Many pwCF taking HEMT are no longer able to expectorate sputum on demand, therefore cough swabs have seen more use in some clinics, despite the lack of confidence in their sensitivity compared with sample type like sputum and bronchoalveolar lavage (BAL).

***Sputum-based culture methods (sputum, induced sputum)***

**Elicitation - Use:** Sputum sampling for culture is a routine method for detection and diagnosis of pulmonary infection. There are multiple indications for use including: pathogen characterisation including resistance profiling, routine surveillance, and for detecting and diagnosing infection in symptomatic people.

**Elicitation - Accuracy:** Sputum is considered a ‘gold standard’ specimen used for infection diagnostics in CF and culture results typically inform clinical management plans.

***“I suppose order [of accuracy] would be cough swabs are next to useless as I say, and sputum is our gold standard.” – CFG2_4***

With the use of HEMT leading to a reduction in expectorated sputum, induced sputum samples are becoming more common. One clinical participant added that sputum induction at times yields secretions that are salivary in nature instead of the ‘classic CF sputum’ and that at times the induction procedure yields no sample altogether despite use of potent strengths of hypertonic saline 

**Elicitation - Acceptability:** pwCF considered expectorated sputum testing to be an acceptable and necessary part of their care, although members of the clinical care team noted that children and teens do not generally like to provide sputum and do not take well to sputum induction.

A participant in a clinical team focus group commented that ‘floating the idea’ of sputum induction would usually be enough to encourage patients to attempt to provide an expectorated sample, which possibly suggests low acceptability of induced sputum, even among adults. On the other hand, it suggests that while expectorated sputum on demand is challenging to obtain, sputum samples expectorated spontaneously outside of the clinic setting can be captured and used.

***“Once we've started floating the idea of induced sputum in clinics, people tend to be able to then cough something up. Sometimes the thought of having to go through an induced sputum does the job.” – CFG2_4***

**Elicitation - Other comments:** One clinical representative stated that following HEMT, the appearance and consistency of sputum had changed for many, with some pwCF equating these changes with being non-productive. One clinical care team participant felt that in this instance pwCF would benefit from receiving education on sputum changes associated with HEMT while being reminded of the utility of sending their samples for testing anyway as many were still expectorating useful samples.

***“What [CF patients] perceive to be sputum has changed, so they're telling you they're non-productive because what they're producing is white, or not as thick and sticky, but it's still sputum. So it's also a bit of education to the patients that actually something that looks fairly innocuous to them is actually still worth sending.” – CFG2_1***

Early detection of problematic pathogens via sinus sampling/upper airway was suggested by participants across the clinical team and pwCF focus groups. Some participants in the pwCF group described experiencing sinus symptoms in the lead up to a lung infection. This was validated by a participant in the clinical care team who said that they had pwCF on HEMT who were experiencing sinus symptoms and that at times they performed sinus sampling at their centre. They added that even where pathogens like *Pseudomonas* are cleared, there is a risk of reseeding via the sinuses

***Bronchoscopy and BAL***

**Elicitation - Use:** Bronchoscopy and BAL was not used routinely, but would be considered where additional information was required. Bronchoscopy is a generally invasive technique, requiring specialist teams and equipment- it is not performed as a first-line diagnostic.

One clinical participant stated that bronchoscopies are more commonly used in cases of suspected or proven Non-tuberculous mycobacterium (NTM) infection due to concerns of impact on lung health. Here, bronchoscopy would be used to inform diagnosis and, or response to treatment.

***“Some children will be referred for bronchoscopy, but we'll have done other things first.” – CFG2_3***

**Elicitation - Accuracy:** No concerns were raised from participants in the pwCF group or clinical team group regarding the accuracy of bronchoscopy. One clinical team participant noted that on the occasions they do utilise bronchoscopy in adults, rarely do they gain additional information from the resulting cultures.

***“Rarely would we do bronchoscopy in adults. And on the occasions that we do … quite often there isn't a lot of microbiology that comes back from that.” – CFG2_4***

**Elicitation - Acceptability:** Participants across the focus groups stated that bronchoscopy was invasive, and acceptability amongst pwCF was poor. Participants in this group said their preference was to avoid undergoing bronchoscopy if possible, and the clinical teams would consider other tests first before deciding to perform a bronchoscopy.

**Elicitation - Other comments:** Bronchoscopy comes with inherent risk associated with the procedure. While those risks are generally low, they do have to be considered when planning clinical management.

***Spirometry***

**Elicitation - Use:** Spirometry, in particular Forced expiratory volume (FEV1) is used routinely for monitoring lung function and general health. Many pwCF have a spirometry device at home, which can be used daily and has utility as an early indicator of infection or exacerbation. Lung function testing can also be performed during routine clinic visits for continued monitoring and may be utilised during periods of infection or exacerbation to determine treatment efficacy.

For acute exacerbations, the goal of treatment is to ensure the patient returns to their baseline FEV1. Continued decline in lung function over time impacts morbidity and mortality. Regular monitoring of lung function is therefore encouraged by clinical care teams, both in periods of health and during periods of acute exacerbations

***“[It is] absolutely the imperative to get the patient back to baseline. We know that in about a quarter of exacerbations, patients do not return to their baseline. And their trajectory over the long term is worse. So it's a real key measure of treatment response. Definitely.” – CFG1_4***

**Elicitation - Accuracy:** These techniques are not typically used in isolation for diagnosis of infection, but do indicate a potential issue that would be followed-up by other diagnostic testing and clinical investigations. Individual lung function measurements are not considered reliable, confidence in these tests comes from repeated measures, where longer term changes compared to a healthy baseline reading can flag decline in lung health or indicate improvements in health.

Some pwCF noted issues with the reliability of spirometry readings performed in the clinic, highlighting that different machines would give different readings, even when used sequentially. They described some machines as ‘temperamental’, requiring multiple attempts to generate a valid reading. This would occasionally prolong clinical visits and cause issues with interpretation of results.

***“It always tells me I've done a bad effort, no matter how hard I'm trying, until I'm purple in the face. What can I do? ... it's quite annoying as the staff don't know what to do about it either. So we're kinda stuck...” – pwCF_1***

**Elicitation - Acceptability:** Spirometry is an integral part of pwCFs clinic visits. Adult pwCF appeared to have accepted the technology as a standard part of managing their life with CF. Clinical staff did not note any particular acceptability issues with spirometry from a patient or clinical perspective.

Regarding home spirometry for general monitoring purposes, participants in the pwCF group offered opposing views on the importance and helpfulness of these tests for monitoring of their lung health. While all of the participants stated they were generally compliant with adhering to treatment regimens and attending clinic visits, some found home spirometry to be too intrusive for them to use regularly. One participant stated that they no longer used their device due to the impact it had on their mental health, largely due to the perceived expectation for them to do the test regularly. Other participants claimed that home spirometry had allowed them to keep on top of their health and gave them a sense of control over their own health and monitoring.

***“I think having the lung function machine at home that you can kind of jump on top of things quickly has been great.” – pwCF_3***
***“I'm the type, I don't like doing regular FEV tests at home… I don't think it was doing mental health any good to be honest. I never use it now. I only do it when I'm in clinic. And I think my mental health is better for it.” – pwCF_4***

**Elicitation - Other comments:** The role of spirometry in CF is primarily in long term health monitoring, although it is also used to monitor treatment response and indicate potential infection. PwCF are encouraged to monitor themselves at home on a regular basis, although compliance varies significantly between people.

***X-ray and CT scans***

**Elicitation - Use:** Clinical team member noted that X-ray and computed tomography (CT) scans were used to obtain baseline images in new, and transitioning pwCF but were not performed routinely. X-rays and CTs are used in part to diagnose infection, but also to rule-out other conditions such as pneumothorax.

***“We do chest X rays, they're not that helpful, unless there's something atypical or concerning going on.” – CFG1_4***
***“In adults, we do chest X-rays … if anyone's more symptomatic mainly, partly to diagnose infection, but also to make sure that it's not anything else as the cause for their symptoms like a pneumothorax.” – CFG2_1***

These imaging techniques are used to inform clinical management and in the case of serial imaging, to evaluate the effectiveness of treatment or interventions.

***“And sometimes it's helpful for us just to get an idea of the trend from […] serial images. So CT we will use quite a lot, not necessarily as a diagnostic.” – CFG2_1***

**Elicitation - Accuracy:** There were no notable concerns elicited from the focus group participants regarding the accuracy of imaging techniques such as X-ray and CT. Generally an X-ray was considered under circumstances where it was indicated to help guide clinical decision making. It was not typically a first-line infection diagnostic tool, and would be used in conjunction with other measures, eg. sputum culture, spirometry, and self-reported signs and symptoms.

**Elicitation - Acceptability:** Participants in both the clinical groups and pwCF group felt that X-rays and CT scans had a purpose in symptomatic, unwell patients, however there was hesitation around frequent use due to concerns around the long terms effects of recurrent radiation exposure.

***“In all honesty, we try and avoid radiology if we can because of radiation. So we'll do it if people are symptomatic or unwell.” – CFG2_1***
***“We now have regular MRIs in place - again to avoid radiation [from repeat x-rays]” – pwCF_1***

***Magnetic resonance imaging (MRI) scans***

**Elicitation - Use:** MRI scanning is used similarly to other imaging techniques such as CT and X-rays, but was less commonly used due to increased cost and lower availability. In fact, NICE guidelines (NG78) for management of CF refer only to use of X-ray ( https://www.nice.org.uk/guidance/ng78). Some participants in the pwCF group stated that their clinic offered them regular MRI scans every six months, however, in the clinical team focus group, some participants commented that they ‘wished’ they could offer MRI scans, but such testing was not feasible for them.

Where it was possible to use MRI scanning, it was considered a valuable diagnostic tool for detecting exacerbations and infections by pwCF and clinicians.

***“There's a lot you can detect in CF with MRI.” – CFG1_4***

**Elicitation - Accuracy:** MRI was considered more accurate than X-rays. There were no concerns raised about MRI regarding test accuracy. It was considered superior to the more frequently used X-ray.

***“I’d love it if we could just do an MRI each time… We do chest X-rays [but] they’re not that helpful.” – CFG1_4***

**Elicitation - Acceptability:** One participant in the pwCF group who’d admitted to having regular MRI scans felt that MRI was much more acceptable to them than X-ray techniques as they were concerned about the effects of radiation exposure following frequent X-ray imaging.

**Elicitation - Other comments:** It is likely that funding allocations between individual centres influence the use of MRI scans across CF centres. This may indicate a degree of regional inequality.

***“MRI. Wow, I'd love it. If we could just do an MRI each time… We're way off that.” – CFG1_4***

***Blood tests***

**Elicitation - Use:** pwCF described the use of blood counts during periods of infection, with particular reference to whole blood cell counts being used as a measure of infection and response to treatment. These measurements would be used alongside spirometry, sputum testing and potentially imaging techniques to support clinical decision making, but would not be used alone as a diagnostic test for infection or exacerbation.

**Elicitation - Accuracy:** No concerns were raised from the pwCF group or clinical care groups regarding the accuracy of blood counts, however, as noted in the use section, this testing forms only part of a series of diagnostic tests.

Some participants in the pwCF group did note that on occasion, the sample of blood provided would be insufficient for the intended tests, which would result in incomplete information, or a possible need for resampling. This was seen as an inconvenience, but was not a major concern as treatment and management decisions would continue without the need for this test.

**Elicitation - Acceptability:** The clinical care groups found blood testing a useful and acceptable addition to the diagnostic and management process. Participants in the pwCF group were divided on their acceptability of blood tests based on their reluctance or dislike of the blood drawing process. Even those who strongly disliked giving blood commented that it was an acceptable option if the resulting test influenced clinical decision making. Upon further questioning, participants in this group generally considered finger-prick testing, as used for glucose monitoring, to be more acceptable than a venous blood draw based on their opinion of the relative discomfort and ease of performing each sampling type.

***“I do quite like a blood test. When your blood cell count goes up, there's a great satisfaction when it's come down again.” – pwCF_3***

**Elicitation - Other comments:** Participants in both the clinical groups and pwCF group stated that they would be happy to see more research in diagnostics focused on using blood to detect infection/exacerbation. One clinical participant commented on the limited utility of c-reactive protein (CRP), adding that an alternative measure would be welcome. As blood is considered a low-invasive, easily collected sample type, with established pathways and protocols for sample collection, transport, storage, and analysis within both primary and secondary care.

***“CRP, I don't think it's going to be that useful, but there might be something better than CRP one day, which could be a home test kit…” – CFG1_4***

***Passive monitoring techniques***

**Elicitation - Use:** While not in clinical use, ongoing trials such as Project Breathe ( https://www.lifearc.org/2023/nhs-trial-uses-artificial-intelligence-to-predict-severe-infections-and-transform-lives-of-people-living-with-chronic-lung-conditions/) involve a range of wearables, digital health devices, and at-home monitoring kits that are expected to inform early detection of exacerbations. Some participants in the pwCF group noted an elevated resting heart rate via their smart watches in the days leading up to symptomatic infection. While this is anecdotal evidence, early feedback from these studies show some promise for the use of continuous monitoring devices in the CF care pathway.

***“Resting heart rate I have found to be a really good indicator of getting an infection before you actually get any other symptoms” – pwCF_4***

**Elicitation - Accuracy:** The accuracy of such digital health and continuous monitoring techniques has not yet been formally assessed. These technologies are not used in the CF care pathway, outside of individual clinical trial use and research experiments.

**Elicitation - Acceptability:** Both the pwCF group and clinical teams were supportive of continuous monitoring technologies. They were considered non-invasive and required the least amount of time and effort from a patient perspective.

A participant in the pwCF group expressed concerns around the potential impact and toll of regular, ongoing data collection on their mental health if a monitoring technology involved frequent data-sharing with clinical care teams and other NHS infrastructures.

***“I'm not sure how comfortable I would be that having access to that [data] all the time. Would it be good for my personal mental health? - To log in and look at it every day?” – pwCF_4***

The majority of participants in this group were very supportive of such technologies and stated that they would feel a greater degree of control over their condition if they could monitor themselves passively and visualise the data generated in their own time.

**Elicitation - Other comments:** While not in clinical use, passive, continuous monitoring technologies appear to offer low impact methods of assessing general health and are used by some pwCF as an early indicator of infection. This does affect the care pathway, as these people are more inclined to seek help earlier in response to changes in parameters such as resting heart rate, even in the absence of other symptoms of infection.

### Interactions between pwCF and clinical care teams

PwCF and clinical team members were asked to describe their interactions with each other to determine the influence of decision making and clinical interactions.

***Stress and anxiety regarding clinic visits***

Some pwCF claimed that their routine clinic visits could be a cause of stress and anxiety on occasion. This was centred on ‘the need to perform’ clinical assessments within the time allocated for their visit. Clinics are an opportunity for staff and pwCF to review their health and management plan, and also to collect routine samples for surveillance purposes, antimicrobial resistance profiling, and obtain up to date spirometry readings. Given the difficulty of expectorating sputum for those on HEMT, and the unreliability of some spirometers, some pwCF reported feeling anxious prior to their clinical visits.

***"I do find clinic quite stressful generally. It’s probably centred around the FEV result... It's just getting a green light that you're okay for another two months is quite stressful" - pwCF_4***

Clinical participants also reflected on the anxiety and stress that clinic visits may incite for pwCF. One care team member commented that the way in which clinic rooms are set up may contribute to this, typically with a clinical team member standing, or elevated on an office chair, with the pwCF in a smaller chair in a corner of the room. This is further compounded by instances where the clinical team member stands over the person while attempting to obtain a respiratory sample to send off for culture. Combined with a busy clinic environment, some pWCF may feel a sense of urgency during their clinic visit.

***“There's definitely something about the technicalities of doing a good cough swab, that mean that you are leering over the person… I think we become clinically immune to how it feels because it's so normal to us… whereas it doesn't feel like that for the person [with CF]” – CFG1_2***

***Reporting of results between clinical care team and pwCF***

There was discussion amongst the pwCF focus group about the reporting of test results. Participants stated they would receive phone calls or messages about their results if the information wasn’t immediately available (e.g cultures taking a minimum of three days), they may also be called into the clinic for some results or if urgent interventions are required.

There were some concerns regarding reporting of results from some pwCF. One noted that they had access to ‘MyChart’, an app where they can view results of any recent tests. They mentioned receiving notification of results far more quickly through this app than from their clinical care team, and occasionally there would be discrepancies between the information accessible via the app and the information obtained via the care team. This appeared to be a discrepancy of detail. One example was an MRI result indicating suspicion of a mild new infection according to their patient record, and their care team relaying there were no concerns from the MRI. It is possible that the care team, having reviewed the report, concluded no further intervention was required, however the pwCF described feeling a loss of trust in their clinical team as a result of this discrepancy.

***“I generally trust their [the care team’s] judgement. But, I will trust it less if I ever feel like I'm not being given the full information.” - pwCF_1***

***Shared decision making***

Participants were asked to describe how decision making was shared between patients and their care team. Both groups described similar experiences. A participant in the clinical team explained that parents of young children with CF, and adults with a late diagnosis of CF, preferred to let the clinical care team lead on decision making as they were still learning about the condition and its management. One participant in the pwCF group with a young child with CF agreed that in paediatrics, decision-making was largely consultant-led, but caregivers were given time to ask questions, discuss the impact of decisions, and process what was happening.

***“It's consultant led, just we're still learning about [their child’s] condition. We’re led very nicely and informed and communicated in the right way and always asked questions - ‘Are you happy that?’ … They give you the time, which I appreciate, our experience has been very positive.” - pwCF_5***

Adults with CF reported greater autonomy and greater say on the decision-making process. The majority of adult CF representatives noted an equal, collaborative approach to decision making between them and their care team. As they learned more about their condition and the signs and symptoms of infection and exacerbation, they were more confident to lead on treatment and management decisions.

***“I find it a very even split… it is very much like a partnership rather than them just telling me.” – pwCF_2***

One pwCF stated that decision making for them was based on whether or not they had experienced the event before. A repeat infection was something they felt they could handle, but they were happy for the clinical team to lead on new infections or treatments that they were unfamiliar with.

For the clinical care team, shared decision making was an important way to ensure pwCF are engaged with their care and understand the impact of their own decisions. The approach for some consultants involves laying out multiple options and explaining the impact or consequences of each, and then leaving the final decision to the individual with CF. One consultant argued that dictating management decisions to teens and adults would likely reduce compliance and adherence to treatment and testing, and should be avoided where possible.

***“I've often got Plan A to Z for some of our patients - This is what we could do. This is what we would suggest you do, and this is why, but it's up to you. And you need to make the final decision.” – CFG2_1***

***The importance of rapport building***

Confidence and trust in the clinical care team appeared to correlate strongly with adherence and compliance with treatment and management regimes in adult pwCF. Due to the chronic nature of the disease, and the ongoing level of interaction involved as part of their care pwCF build a close bond with their care team. As such, most participants felt they were ‘on par’ with many of the clinical care team regarding knowledge of CF.

***“Quite often, you know a hell of a lot more about CF than they do [trainee doctors] … I think after having CF for a long time, apart from those top consultants, you'd know more than almost anybody else.” – pwCF_4***

Building familiarity and rapport with the CF team was particularly important for pwCF with unusual symptoms or specific needs. Participants in the pwCF focus group commented that they all experience CF differently, and it takes considerable time to understand their particular requirements. Thus some stated they would prefer to delay or re-arrange their clinic visits if they weren’t able to be seen by their usual, preferred clinical team.

***“Trainee doctors on their own, you just haven't got the relationship with them. And it takes a long time with CF, to learn about you and for you to learn how they work, and how best to get on.” – pwCF_4***

***Impact of managing CF on lifestyle***

Multiple pwCF noted the impact that managing their CF had on their life, with one describing it as ‘another full time job’. There was discussion amongst the group about the burden of diagnostics performed at the clinic and the shift towards repeated measure tests at home (such as spirometry). Combined with on-going complex treatment regimens which for some involve using nebulised therapies multiple times a day, participants believed that managing their CF interfered with their daily lives.

***“It's like having a job, and I do my treatment. And I go and see my ‘colleagues’ at clinic. It's just habitual… But now it's the personal life too…By putting all of this stuff into data all the time and having someone watch you, it's no longer something you can separate yourself from.” – pwCF_1***
***“When she’s on four nebulizers a day plus other stuff, you go [sigh], then it becomes overwhelming” – pwCF_5***

The parent of a young person with CF added that the clinic visits were particularly disruptive to their lives and their child’s learning. They understood the need to visit the clinic, but claimed that the care team were usually very busy, and the clinic would be running late. Additionally, the CF team would use the opportunity to obtain other clinical measurements which would normally be taken at a routine visit like, height, weight, and physical checks. These would increase the clinic time, adding to the parent’s anxiety for their child’s health.

***“Even just a mundane test, it's not a case of you pop in, you're done within 30 seconds… It becomes a whole mini-review… It shouldn't take three hours to get a cough-swab done… Just tell me as quick as possible, what we need to do” – pwCF_5***

One adult with CF had removed themselves from clinical trials due to the added burden placed on them to repeatedly perform specific tests or attend extra clinics for additional measurements. Another supported the statement, adding that frequent testing and referrals would impact their adherence to treatment and monitoring.

***“I took myself off [a trial] after six weeks… every day, you had to do all these things. I'm actually quite compliant [normally]. And it panned my compliance.” – pwCF_2***
***“That extra burden… As much as it's going to help you, if you don't feel like you need the help at the time, it's easy to forget to actually do it. So, yeah. It's a real balancing act.” – pwCF_6***

### Unmet needs in CF infection and exacerbation

***Monitoring and re-testing in response to treatment***

In paediatric clinics, participants from the clinical team and pwCF focus group noted that cough-swabs and cough-plates were used very frequently in instances where an infection was suspected, and following completion of treatment to determine efficacy.

***"[paediatrics] They're pretty quick to cough-swab when needed, and always cough-swab after treatment" - pwCF_5***

In the adult setting, there was less focus on re-testing or monitoring during periods of infection. Some pwCF stated that once they had started a course of antibiotics, no further tests were performed during or after the course, with decision making for escalation or de-escalation of treatment guided by clinical signs and symptoms.

***"[adult clinic] They didn't run any other tests" - pwCF_4***

Members of adult clinical care teams expressed the need for determining treatment response and treatment efficacy, citing concerns of overtreatment. Clinical focus group members felt that benefit could be derived from shorter antibiotic courses in some instances, and determining effective treatment duration would support antimicrobial stewardship.

***“I think a massive unmet need is knowing how long to treat. We're almost certainly very frequently overtreating, which is obvious that it has consequences - to the patient, to healthcare system, to the wider bacterial resistance out there in the community, is a really hard nut to crack.” – CFG1_4***

Empiric antibiotic use is standard practice, as the reference standard culture techniques take at least three days to provide actionable results, or longer for pathogens like NTM. Clinicians stated that for an exacerbation, they will treat empirically, and with the current standard of diagnostics available, this practice is unlikely to change.

***“It's pretty unusual for us to wait for the sputum in an exacerbation setting… When were not sure what’s going on, then we might wait for the sputum samples, but if someone's clinically unwell, then you treat them anyway.” – CFG2_1***

***Equity of care***

Both the clinical care team and pwCF noted issues with equity of care across the devolved nations and within regions of the UK. PwCF described differences in their care pathways, access to diagnostics and interventions, responsiveness to need, and frequency of clinic visits.

***“I just wanted to say that the difference between CF centres even now is great. When I speak to other patients, some of the things that patients experience, I just think - my goodness!” – pwCF_2***

Clinical representatives noted differences in local Trust guidelines, funding streams and allocation of funding, availability of services and staff, and access to treatment options all contributed to inequity of care for pwCF across the UK NHS, with one clinician stating an individual’s care was largely dependent on their postcode.

***“Yeah, my long experience working in the NHS is it's horrible to hear things like … an individual's care will depend on what postcode they're in, sadly, that happens ... But we don't want it to happen.” – CFG2_2***

A participant from the microbiology lab noted significant differences in the preparation and analysis of samples, particularly cough swabs and sputum. This difference was attributed to the size and capacity of the laboratory, as well as individual Trust guidelines. They suggested that additional guidelines for laboratory methods would improve consistency across the UK.

***“Everybody seems to do things a little differently depending on where you work. Some people do 21 day culture for fungi, and we are doing five days, and well, 10 must be better than five, I think there's not consistency between the laboratories … For a patient, the quality of care will depend on where you go. And that could be also down to the laboratory who process your samples” - CFG1_6***

Equity of care was also linked to funding structures between the four UK nations. This also extended to availability of drugs and other interventions across the UK nations, which is dependent on inclusion of these interventions in national formularies in different parts of the country.

***“CF care is funded differently in each of the four UK nations. The way that funding splits between direct clinical care, versus microbiological care, versus radiological is complicated... so it's almost impossible to know.” – CFG2_1***
***“The postcode lottery is also about drugs coming on to formularies in different parts of the country. We can prescribe certain things to English patients that we can't prescribe to Welsh patients.” – CFG2_1***

***More acceptable methods of sampling***

The clinical care teams, particularly physiotherapists involved in the collection of sputum samples, stated that sputum sampling was not well received by some pwCF, particularly with the increased use of HEMT, where those on HEMT are increasingly non-productive, and must undergo a less tolerable induced method of sputum sampling.

***“We need more acceptable testing... I think nirvana is definitely a test where we don't need sputum… There are a number of people where we don't have sufficient samples to be confident about what's going on. And that's not because there's no sputum there. That's because it's not a comfortable way to test.” – CFG1_2***
***“The biggest thing we've got to think about is an alternative to sputum samples” – CFG1_5***

Collection of induced sputum has impacted clinic times, as the process takes longer and increases the burden of time for the physiotherapists. The reduced acceptability of induced sputum sampling was also highlighted across all focus groups - the potential risk is that fewer infections are detected due to fewer high quality samples being collected.

***“Time is quite a big issue from a physio perspective. [With modulators] we're spending up to an hour and a half, with people to develop the induced sputum.” – CFG2_4***

Cough swabs, while generally more acceptable to pwCF, were less acceptable to clinicians due to their poor accuracy, in particular, low negative predictive value (NPV). Negative predictive value relates to the probability that following a ‘negative’ test result, that individual will not have that condition. A poor NPV means that a negative test is potentially incorrect, and the patient may in fact have the condition being tested for, despite a negative result.

***“Cough swabs, generally are a good positive predictor, but not a great negative predictor. If it's clear, then I don't think we're confident it's clear … Is that clinically relevant?” – CFG1_2***

***Faster microbiology and bacterial culture testing***

For the clinical care team, the time to results for culture-based diagnostics is usually too long to consider delaying the use of empirical antibiotics. Treatment decisions are based primarily on clinical symptoms and choice of antibiotics guided by results from previous cultures. Mycobacteria in particular takes weeks to grow sufficiently in culture for analysis.

***“We'll be making antibiotic treatment decisions before we see acute samples. So we might be able to get acute virology back very quickly, but in terms of microbiology, bacterial culture, we will be making the antibiotic decisions based on previous samples, not the acute ones at that stage.”* - CFG2_1**

In addition to the culture time, antibiotic susceptibility testing also takes time. In acute situations, this delay is unacceptable for clinical decision making, further emphasizing empirical antibiotic use.

***“Current culture based techniques are slow. It can take ages to get any kind of microbial sensitivity for what it's worth. So I think, better, faster identification of worrying mycobacteria, like mycobacterium abscessus, would be helpful.” – CFG2_2***

Changes to the care pathway, and a move towards postal-based sputum samples was preferred by some participants in the pwCF group to samples collected in clinic, however clinical participants flagged some issues around transporting the samples. One clinical representative noted that they had observed more issues with contamination and false-positive cultures following a period of delays within the postal system. Transit time of posted sputum samples affected the quality and resulting accuracy of culture-based diagnostics.

***“We've looked at our postal sputum results over the last two years and compared it to a much smaller percentage of postal sputum results before the pandemic. And without doubt, delay to lab - transit time, directly correlates with the appearance of unusual bacteria and unusual Pseudomonas species.” – CFG1_4***

Clinical representatives agreed that a rapid alternative to culture-based diagnostics would be valuable to delivering timely care. Rapid detection of non-Tuberculosis mycobacteria and *Pseudomonas* were explicitly referenced as cases where earlier, faster diagnosis would be of value in the care pathway, particularly if there was a quantitative element to the output.

***“It’d be really nice to have rapid, faster than culture, diagnostics, with quantitative elements.” – CFG2_2***
***“Certainly in TB, they've moved really far forward with this now [rapid testing], so it would be quite nice to see similar with NTm.” – CFG2_1***
***“With a new Pseudomonas, you want to action it a bit quicker, you don't want to be waiting around in the same way you can afford to in other situations.” - CFG2_4***

***Communication and integration of services***

Laboratory staff involved in analysing samples from pwCF described a desperate need for greater communication between laboratory staff and clinical staff. One microbiologist noted that they had been given a seat at their local multi-disciplinary team meetings, which had improved communication and allowed the clinical team and laboratory staff to discuss clinical cases in greater detail. This was not standard practice for many CF centres though.

***“From a laboratory point of view, we need to be more communicative to the team. So you’ve got to have that really tight link with the laboratories.” – CFG1_6***

Another microbiologist stated that samples frequently arrived with inaccurate information, or inappropriate fields completed on accompanying forms to inform the laboratory of what the sample is, and what is required from it. They stated that this leads to inappropriate handling of the sample, failed tests, and potentially incorrect reporting of results.

***“Accurate information initially. And this is particularly significant for CF sputum specimens that turn up without the relevant information, the inappropriate culture media will be put on, and reports and everything else following on from that will be delayed.” – CFG2_6***

Improvements to education and training of laboratory staff for the handling and identification of unusual bacterial and fungal infections, particularly those more commonly found in CF, was also considered an unmet need for laboratory staff, along with greater, more consistent standards for the testing of samples. Laboratory staff had noted that non-specialist laboratory staff were more likely to misidentify strains isolated from a pwCF and may inaccurately report findings that clinical staff use to inform their clinical decision making.

***“Improving the standards of education within labs, consistency of what we do, it's just not consistent. And I think that could be improved by education and discussions and forcing standards on [laboratory staff]” – CFG1_6***

### Stakeholder preferences in TPP characteristics

All of the focus groups involved a TPP characteristic preference and ranking exercise. After an introduction to the topic, each participant in turn was given time to think about what characteristics they felt were most important when considering current and future diagnostics in CF infection and exacerbation.

***Preferences for pwCF***

**Accuracy and confidence in testing** – For participants in the pwCF group, accuracy of a test and the confidence they had in a test were discussed simultaneously. PwCF expressed concerns about at-home testing and ensuring that they were performing the test correctly. Some of them alluded to COVID-19, and the proliferation of at-home nasal-swab lateral flow devices. Reliability is of particular importance to pwCF, as multiple participants stated that if a test failed, it would usually mean that they’d have to re-attend clinic in order for the sample to be collected again, or it would delay the time to results which would inform their treatment. With many pwCF becoming less productive secondary to HEMT, the importance of reliability of testing on increasingly hard to get samples is significant.

***"At the end of the day [the best test] is whatever’s going to be most reliable." - pwCF_6***

Regarding test accuracy, participants noted higher accuracy would be preferable as diagnostics for infection and exacerbation in CF are used to inform treatment and management plans.

***"The main thing is accuracy, I suppose. Because what's the point in doing the test, it doesn't give you a good result." - pwCF_4***

**Time to results** – Participants in the pwCF group stated one of their main unmet needs involved faster time to results, particularly for culture-based methods.

***"Speed of result is top, definitely." - pwCF_2***

Clinical care team representatives outlined time to results for culture-based methods are typically 2–3 days for a ‘negative’ culture, 3–5 days for a ‘positive’ culture of a fast-growing pathogen like P *seudomonas*, but could take 6–8 weeks for some pathogens like NTM and some fungal species can be up to 28 days (e.g. *Exophiala*) (clinical focus group 1). New infections with either of these bacteria can be a health emergency for a pwCF, usually requiring a treatment regimen intended to eradicate the pathogen. Participants in the pwCF group stated that the delay in time to results generally means they are prescribed antibiotics empirically, or treatment decisions are delayed, thereby increasing the risk of the infection worsening before an intervention takes place.

 A faster time to results was considered by pwCF to lead to earlier intervention, and reduced risk of complications.

***"I want to get on it as quickly as possible, rather than it escalates into something that I know is going to be like three, four weeks stay in hospital" - pwCF_3***

Many participants in the pwCF group claimed their symptoms would start in their sinuses prior to signs of infection on the lungs. They added that using samples from the sinuses or upper airways could be a way of detecting problematic pathogens before they cause a lung infection.

***"If it can be simplified, and it can be sped up… waiting for it to be on the lungs is kind of a bit too late" - pwCF_5***

**Acceptability and convenience of diagnostics –** While there were mixed views on which specific diagnostic tests were deemed acceptable, there was a preference for convenient and minimally invasive tests.

***"Unpleasantness… Because some tests are pretty horrible, and I would have to be pretty ill to agree to have them" - pwCF_4***

As discussed in other sections, pwCF preferences for rapid testing was in part linked to the significant impact that CF has on their lives. They wanted to ensure that any tests they did were useful, quick, and had some clinical impact. Many wanted to ‘get on with their lives’ and not have to think about their condition more than necessary.

***"That's my experience, you just want to get on, and live your life" - pwCF_5***
***"You're not going to do it [testing] every day if you feel like you don't have to." - pwCF_6***

At-home testing was particularly preferred by the majority of pwCF. The opportunity for postal sputum, was seen as convenient and time saving, and also minimised the risk of cross infection associated with the hospital setting.

***"The things you can do at home, that can take out the onerous elements of going to the hospital...Home tests, results come in quicker" - pwCF_5***

***Clinical care team preferences***

**Accuracy, sensitivity and specificity -** Multiple clinical representatives stated that accuracy was more important than time to results, and that they would prefer to wait a longer for a more accurate test if the alternative was a quicker test that required additional follow up testing due to an inferior accuracy.

***“Speed is important, but I'd rather have a test that takes 24 hours, and I can act on it, than one that takes one hour, but I don't know if it's wrong or right.” - CFG2_1***
***“I think that speed sounds like a great idea, but if you then have to do another test to be certain of the result, perhaps you can wait a little longer [for an accurate test], as long as it's not too long, that's the most important thing.” – CFG2_5***

Clinical representatives noted that high accuracy, both in terms of specificity and sensitivity, would be important if a diagnostic is to inform clinical decision making.

***“Sensitivity and specificity really needs to be thought about. And these need to be very high for both to be quite honest.” – CFG1_4***

Some clinical representatives suggested that sensitivity of the test would be more important than specificity, claiming that over-treatment (in part linked to false positives) may be less severe for a patient than missing an infection (due to false negatives).

***“High Sensitivity, high specificity, you don't want to have false positive and even worse, false negatives… it's easier to undo something, than missing something and then trying to catch up. A test has to be sensitive.” – CFG1_1***

Other team members argued that false positives should be avoided, due to the anxiety this can cause for patients, particularly with pathogens like Pseudomonas.

***“It is important that we don't see so many false positives, because that causes a huge amount of anxiety for patients. A false positive Pseudomonas would be huge for a patient who hasn’t had Pseudomonas previously” – CFG2_1***

One care team representative offered preferred accuracy values, stating that 70% accuracy would be unhelpful, and 95% accuracy would be reasonable for them to take actions based on the result of that test.

***“There's no use having a test that's 50% of the time accurate. I'd like it to be accurate 95% of the time. There's no point saying we only want it to be accurate 70% of the time, otherwise it's just not very helpful.” – CFG1_5***

Justification for high test accuracy was provided by one clinical representative. They noted that treatment decisions, including lung transplantation was contingent on the person’s microbiology results. False results would severely impact the patient and their management.

***“You don't want to reassure someone that everything's absolutely fine. And then they go for their transplant assessment, and hey presto, they've grown something brand new that you've missed. But also we don’t want to be spotting things that are irrelevant or not there, because we already have patients who are highly treated, we don't want them to be over-treated needlessly.” – CFG1_2***

**Patient acceptability –** Clinical team members considered patient acceptability of significant importance to the overall effectiveness of a novel diagnostic. Compliance is required for optimal management and improved outcomes, and patient acceptability of interventions is a key driver of compliance and adherence.

***“If we aren’t focused on what's acceptable to people with CF, then it's impossible to implement them. Everything we do is by collaboration and consent with people with CF.” – CFG1_2***

Along with driving compliance, physiotherapists described the impact that acceptability has on the time spent collecting a sample, and the impact that this has on the relationship and rapport built with the patient.

***“If you spend a lot of your time having to persuade and almost coerce people into doing that test, then it's not helpful, especially in an area where you do need that long term relationship with people.” – CFG1_2***

**Time to results –**An acceptable ‘time to results’ is vital to informing timely clinical decision making, particularly in the setting, or in cases where pathogens like *Pseudomonas* are detected for the first time and eradication therapy is required.

A faster time to result was considered to drive informed and appropriate clinical decision making.

***“It is speed of diagnosis really, about what they might be growing, because we do treat based on their symptoms.” - CFG2_3***

Increased reliance on induced sputum collection has impacted the time and efficiency of CF physiotherapists, a faster time to result should therefore also consider the time it takes to collect the sample material required and the time to transport the sample to the laboratory.

***“It used to be that you'd hand someone a pot on the way into clinic, it would be full by the time they left. Whereas now, we're spending quite a significant amount of time… to get not even the same sort of sample you would have had previously.” – CFG2_4***

Clinicians also considered the time involved in analysing a sample and performing a test, providing examples of cases where non-24 hour services have resulted in delays to clinical management. This delay resulted in a patient continuing a course of antibiotics in the absence of information validating the appropriateness of the therapy.

***“A patient dropped off sputum on Friday, and that patient is at home on an antibiotic, but it might not be the right antibiotics make them feel better. [With a faster time to result] he could have a different antibiotic, if I just knew what we were treating.” – CFG1_3***

**Clinical relevancy (impact on treatment decisions) –** Clinical care teams claimed that a test would only add value to the care pathway if it was utilised in such a way that it could impact clinical decision making.

***“So what we teach our medical students - the ideal test is one that will change your clinical management. And that's the key isn't it?” – CFG2_1***
***“Having got the result of this test, am I further along knowing whether or not I need to change this person's treatment?” – CFG2_2***

When questioned further on the topic, one clinician noted that diagnostic tests should inform antibiotic strategies in order to be of clinical benefit as empirical use of antibiotics is commonplace in CF management, an issue noted by both clinical care staff and pwCF.

***“It's really about our antibiotics strategy, so not just the result, but what you actually do as a consequence of that result, because our standards of care are woefully out of date.” – CFG2_1***

**Integration into the clinical care pathway (staff acceptability) –** Disruption to the current care pathway when integrating a novel diagnostic can significantly impact staffing arrangements, cost and clinical decision-making processes, potentially delaying clinical care, or resulting in poor functionality of the care pathway. Clinical representatives stated that it would be important for a novel diagnostic to fit within the current care pathway with as little disruptions as possible.

***“[You need a test that] can realistically fit into the pathway. If [a test is] not acceptable to us, it is not going to work. Something that is acceptable to the team in terms of all aspects - size, cost, ability to use it, how complicated it is to use, and training needs for people to use it.” – CFG1_2***

One clinical representative stated that the logistics of integrating and delivering a novel intervention is often overlooked by scientists and researchers, and would need additional thought to be successfully integrated into clinical practice.

***“Definitely in terms of drug development, when you talk to some of the scientists, they're thinking about how they will deliver it, but not the logistics of getting the machine that it connects to, and who pays for it, and who pays for the servicing… And these are things that are very complicated, and do require a bit of thought in terms of translating something new into actual clinical practice.” - CFG2_1***

### The future of CF care and diagnostics

Participants in the pwCF and clinical focus groups provided opinions on what they would like to see from novel diagnostics and care pathway changes in CF.

**Improved sample usage –** The clinical care team were keen to see greater use of existing and easy to obtain sample material. With sputum becoming increasingly difficult to obtain, being able to perform multiple tests on a single sample, was considered an important step in the advancement of CF diagnostics. An example of this in practice is with viral testing, where some CF centres require multiple samples for an array of tests, while other centres use one swab for multiple assays. Using a single sample across multiple assays was considered a convenient approach and one viewed as less burdensome for pwCF.

***“In some centres you have to provide multiple samples for a viral swab, whereas in other centres they say send us what you've got, and we'll just test it for everything we possibly can. So I think there's something about reducing the number of samples that we need to send.” – CFG1_2***
***“If you've got lots of little tests that give you a bigger picture, can you still take one blood sample or one sputum sample or one whatever sample? Or do you really need to take five or six different ones? I imagine the acceptability for some will depend on what the answer to that is.” – CFG2_5***
***“Anything that reduces the number of sputum samples we need, or reduces the need for it…” – CFG1_2***

**Better staff training for assessment of CF cultures –** One microbiology representative highlighted that laboratory staff unfamiliar with handling CF samples would frequently miss atypical or unusual pathogens. With CF centres operating differently in terms of laboratory staffing and training, sample handling may vary between the centres. The representative expressed a desire for greater training and awareness amongst laboratory staff in the detection of CF-relevant pathogens, as well as further guidelines on culturing methods and restrictions on which staff members can report on microbiology results.

***“When you're a scientist and reading CF cultures, if you don't have experience, it's very easy to miss an atypical pathogen. [In some CF centres] you'll have trainees on there, and you can guarantee they're going to miss some pathogens. And we know this is a training issue.” – CFG1_6***

**New biomarkers for exacerbation and infection –** Clinical care representatives stated that they would like to see novel biomarkers for CF infection and exacerbation, and greater research into optimal duration of treatment.

One clinician argued for a biomarker to identify ‘risk of deterioration’ aiming for ‘something better than CRP’ that could be performed at home to distinguish bacterial or viral infection rapidly. They added that understanding if a someone is experiencing a viral or bacterial exacerbation earlier would support antibiotic stewardship and expose fewer pwCF to ineffective treatments (eg. a case of a viral infection and empirical antibiotic use).

***“There might be something better than CRP one day, which could be a home test kit, that would say actually, you are about to exacerbate, now you need antibiotics - seven days… What you want is an all-encompassing biomarker that says this person is at risk of deterioration, and it's highly likely it's a bacterial cause for that deterioration, and they need to treatment.” – CFG1_4***

Other clinical team members also challenged the standard 14-day antibiotic course, and considered a biomarker for detection and monitoring would allow for tailored antibiotic use for the optimal duration. One clinical care participant added that they had shortened the duration of antibiotic treatment to 7–10 days for many of their patients.

***“[We need] other biomarkers to help us to monitor infection… a greater, more efficient way of doing that - if we're not going to be able to grow things or get sputum, what are the biomarkers can we use to monitor and diagnose infection?” – CFG1_5***
***“If we had a biomarker that can tell us there's an infection and a biomarker to tell us there's no longer an infection, and we can just give three days of treatment in this instance, that's a lot better than giving 14 days of standard.” – CFG1_4***

**Further research into digital health –** Both pwCFs and clinicians referenced ongoing clinical trials including ‘Project Breathe’ ( https://www.lifearc.org/2023/nhs-trial-uses-artificial-intelligence-to-predict-severe-infections-and-transform-lives-of-people-living-with-chronic-lung-conditions/), which utilises a number of digital health technologies including Bluetooth enabled devices and smart watches to collect information on basic health measures. Spirometry, resting heart rate, and temperature are measured, and will be later analysed with machine learning techniques to determine if ‘passive’ monitoring can flag potential exacerbations and infections before clinical signs and symptoms are evident. Such techniques were considered generally acceptable and low impact for participants in the pwCF group, and may support earlier decision making for CF care teams.

***“We've issued a lot of Fitbits, Bluetooth connected spirometers, Bluetooth connected weighing scales, which feed into a portal… So it's interesting. If it does predict - great, it'd be brilliant… because it is just in tune with what everyone likes to do - wearing watches and devices and things.” – CFG1_4***

**Determining colonisation from infection –** An outstanding clinical and research question concerning the separation of colonisation from acute exacerbation was discussed in the clinical focus groups. Clinical experts suggested further research into the microbiome in pwCF with and without a chronic infection would lead to greater understanding of the difference between normal flora, colonisation and acute exacerbation and help better inform clinical decision making.

***“What is normal flora in a person with CF? Because I think that's a massive unknown, and would help us make decisions when we get results later.” - CFG2_2***
***“The microbiome and understanding that properly… It's amazing research and science. If we could crack that, you could correlate that with the risk of exacerbation and therefore, the need for treatment.” – CFG1_4***

One clinical team member argued that detection of pathogens alone is not sufficient to determine treatment, and that newer, genetic techniques may be overly sensitive, capable of detecting pathogens that would not actually result in clinical deterioration. Combining pathogen detection with a biomarker of disease activity and or severity may address concerns regarding oversensitivity and provide additional clinical context for better decision making.

***“Particularly with the microbiome stuff, there's a lot down there [in the lungs] that we don't see. And then we randomly do see it in culture and treat it. And actually it was there all along. If I put my inflammation hat on, is bacterial diagnosis alone is sufficient, or do you actually want some idea of disease activity as a consequence? [This] might help you understand whether it's just sitting there or is actually driving any active process at the time.” – CFG2_1***

**Therapeutic drug monitoring-** Timely turnaround of drug levels for therapies which require therapeutic drug monitoring to enable safe and effective drug dosing.

**Route of drug delivery -** While not related to diagnostic tests, participants in the pwCF group shared some insights on the use of nebulised antibiotics delivered via mask. Participants suggested that more research would be welcome to determine the efficacy of nebulised antimicrobials delivered via mask rather than mouthpiece, particularly in the context of sinus disease. Participants reported experiencing sinus symptoms in the lead up to a pulmonary infection, and felt in such instances nebulised antibiotics delivered via mask should be considered.

***“My team suggested IVs and I actually said can I first try inhaled antibiotics through a mask, which I've never had before, I've only ever done it through a mouthpiece. Knowing that everything starts in my sinuses, we tried it. And it was a really quick culture conversion to negative. So I'd love if that could be further explored in research... I really feel like that should be encouraged more.” – pwCF_1***
***“I think the mask is actually better. Because you can kind of hit the lungs better… You're still inhaling and it's still going into your system, which is essentially what you need.” – pwCF_3***

## Discussion

This body of work represents the views and opinions of pwCF and clinical care team representatives on the current CF care pathway; the acceptability, indication for use, and role of current diagnostics used in the UK NHS for detection of infection and exacerbation. These focus groups highlighted unmet needs in the existing CF care pathway, some of which may be addressed through the introduction of novel diagnostics, including the introduction of tests for detecting, diagnosing and monitoring infection and treatment response.

Preferences for test characteristics, along with a rudimentary ranking exercise, elicited key diagnostic test characteristics of importance to clinical and pwCF representatives. Some of these were shared between pwCF and clinical team representatives, namely ‘acceptability’ to pwCF, ‘test accuracy’ and ‘time to results’ and could be seen as fundamental criteria for the successful adoption of a test into the CF care pathway. However, successful adoption also hinged on navigation of divergent care pathways and funding models between regions of the UK, leading both groups to identify a lack of equity in CF care. Other characteristics of interest included the burden a test would place on pwCF, convenience, and the utility of the test within current standards of care.

Participants from the pwCF and clinical team focus group provided opinions on the future of CF care and diagnostics, highlighting several areas where they would like to see more progress, research or training. This exercise revealed demand for novel biomarkers for infection and exacerbation, optimal use of routinely collected samples, and further research into the lung microbiome of pwCF. Advances in digital health, additional training for laboratory staff on sample handling and a desire from pwCF for early management of sinus infections were all discussed as areas where the care pathway, or care experience could be improved.

Synthesising and analysing this feedback simultaneously reveals a preference for less invasive, convenient and rapid testing for exacerbation and infection in CF. Key pathogens of concern due to their impact on health outcomes were, strains of *Pseudomonas aeruginosa* and NTM. In paediatrics *Haemophilus Influenzae* and *Staphylococcus aureus* were also pathogens of concern. More rapid methods of detecting these key pathogens would likely have a positive impact on clinical care and inform earlier decision making. Participants from the pwCF and clinical team focus groups highlighted the need for suitable alternatives to sputum, favouring less invasive, easy to collect sample types with good clinical utility. Blood-based assays and breath condensates were of notable interest in this regard. Participants across all groups emphasised the need for novel biomarkers for the early detection and diagnosis of infection and exacerbation to facilitate better informed antibiotic selection and move away from traditional empirical approaches. Participants felt that with the widespread use of HEMT, the basic health measures of pwCF have improved, and as such, less reactive, and more informed decision-making on care and management should be achievable. This was supported through the TPP characteristic discussion exercise, which revealed that under non-acute circumstances, test accuracy was largely valued over the time to results for a clinical test.

Both pwCF and clinical participants highlighted the importance of human-factors in the management and treatment of infections and exacerbations, including emphasis on shared decision-making, the potential anxiety caused by appointments, the burden of CF management and the how a good rapport with CF specialists contributes to management of infections.

This work underpinned the foundations of a formal TPP elicitation exercise and indicated a number of shared preferences amongst clinical staff and patients. The priorities identified here contributed directly to TPP content and further stakeholder engagement in the subsequent stages of the project.

### Study limitations

While the depth and quality of the information gathered in this report is valuable, the small sample numbers involved means that the conclusions drawn in this report may not be representative of the UK NHS or CF population as a whole. Indeed, the pwCF who interact and volunteer in such activities as those utilised in this body of work may not be representative of the general CF population. It is likely that these pwCF are more informed, more interested in medical research, and may be more responsive and compliant in their own management of their condition. Equally, clinical representatives may have research interests or other motivations which drive them to take part in this work and thus, their opinions and conclusions may not be representative of the wider clinical body. The authors themselves are not clinical and do not work exclusively or routinely in the area of CF, and therefore this may have influenced our interpretations of the clinical and pwCF focus group data. However members of the external advisory group for the TPP development did include pwCF and clinical care teams experienced in CF management, both of whom reviewed all proposed TPP content.

The nature of focus group-based elicitation means that some representatives may overshadow other members of the group, and breadth of opinion may be lost under more generally accepted opinions. This is addressed to some extent by the methodology team directing questions to specific representatives and ensuring opportunities to comment on the conversation topics were available, however it is possible that some representatives may have refrained from sharing their opinion on some topic areas in front of the group.

Not all of the care staff involved in the care of pwCF were included in the focus groups, and only one or two representatives were involved from each discipline, further biasing the opinions gathered.

Despite these limitations, the conclusions drawn from this work formed a valuable step in the generation of a patient-focused TPP for CF infection and exacerbation diagnosis. The methodology team aimed to address the limitations discussed here in their approach to the formal TPP elicitation exercise.

## Conclusions

These focus groups offered rich and detailed insights into the opinions of clinical staff and pwCF alike on the current landscape of diagnosis and management of pulmonary infection and exacerbations in CF.

This work reveals a preference for less invasive, convenient, and rapid testing for detecting and diagnosing infections and exacerbations in pwCF. Key pathogens of concern were those with a high risk to pwCF, such as strains of Pseudomonas aeruginosa, and NTM. Early and rapid detection, particularly of these key pathogens, would likely have a positive impact on clinical care and inform earlier clinical decision making.

In the era of HEMT, participants across all groups highlighted the need for suitable alternatives to sputum, favouring less invasive, easy to collect sample types with good clinical utility. Blood-based assays and breath condensates for example, were of notable interest in this regard. Participants across all groups emphasised the need for novel biomarkers for the early detection and diagnosis of infection and exacerbation, better informing antibiotic selection and a move away from traditional empirical approaches. Amongst clinical representatives, test accuracy was largely valued over the time to results for a clinical test in a non-acute setting.

These focus groups and the evidence generated underpinned the foundations of a formal TPP elicitation exercise and indicated a number of shared preferences amongst clinical staff and pwCF that were taken forward and addressed in the subsequent stages of this project.

## Ethical approval and consent

The focus group with persons with CF was authorised as part of a service evaluation approved by Newcastle upon Tyne Hospitals Trust in May 2022 prior to the focus group taking place (project number: 13687).

This remainder of this body of work is registered through the Integrated Research Application System (IRAS) - Reference number: 316276. Ethical approval was granted through Favourable Opinion of the Cambridge Central Research Ethics Committee and the project received a letter of approval on 18th October 2022 to cover interviews and focus groups with NHS clinical staff.

All identifiable information collected during the elicitation process has been anonymised prior to reporting. For recording of the focus groups, consent was confirmed verbally by the participants prior to starting the focus group elicitation exercise and witnessed and recorded on the consent form by a second researcher. Verbal consent was thought to be most appropriate, due to the focus groups being conducted remotely and placed least burden on participants. The collection of verbal consent was approved as part of the above-mentioned IRAS application and in the approval for the service evaluation. 

## Abbreviations list

**Table:** 

Abbreviation	Definition
AMR	Antimicrobial resistance
BAL	Bronchoalveolar lavage
CF	Cystic fibrosis
CFTR	Cystic fibrosis transmembrane conductance regulator
CNS	Clinical nurse specialist
CRP	C-reactive protein
CSLD	Chronic suppurative lung disease
CT	Computed tomography
ETI	Elexacaftor/Tezacaftor/Ivacaftor
FEV1	Forced expiratory volume
HEMT	Highly effective modulator therapy
IRAS	Integrated research application system
MDC	Manchester Medicines Discovery Catapult
MRI	Magnetic resonance imaging
NICE	National Institute for Health and Care Excellence
NIHR HRC	NIHR Health Technology Research Centre
NIHR Newcastle MIC	NIHR Newcastle MedTech in vitro Diagnostics Cooperative
NTM	Non-tuberculous mycobacterium
NPV	Negative predictive value
PPIE	Patient and public involvement and engagement
PPV	Positive predictive value
pwCF	People with CF
TPP	Target product profile

## Data Availability

Figshare: Cystic Fibrosis Target Product Profiles: Focus group data (clinical and people with CF)’ are available at Newcastle University, 10.25405/data.ncl.26565871 ( Howe *et al*., 2024b). This project contains the following underlying data files: Data file 1- Clinical Focus Group 1 transcript Data file 2- Clinical Focus Group 2 transcript Data file 3- Patient focus group transcript Data are available under the terms of the Creative Commons Attribution 4.0 International (CC-BY-4.0). Newcastle University Figshare: Extended data for ‘Eliciting required and desired diagnostic test characteristics to develop patient-focused target product profiles (TPPs) for diagnosing infection and exacerbations in cystic fibrosis.’, https://doi.org/10.25405/data.ncl.26477263.v1( Howe *et al*., 2024a). This project contains the following extended data: Data file 1- Clinical focus group topic guide Data file 2- pwCF focus group topic guide Data file 3- Diagnostic TPPs lay summary Data file 4- Information for clinical team focus group Data are available under the terms of the Creative Commons Zero “No rights reserved” data waiver (CC0 1.0 Public domain dedication).
