# Supplementary material for: Unmet diagnostic needs in cystic fibrosis infections and exacerbations: focus groups to inform target product profiles (TPPs)
Source: NIHR Open Res. 2025 Dec 2;4:81. Originally published 2024 Dec 24. [Version 2] doi: 10.3310/nihropenres.13674.2 (PMC12598273; doi:10.3310/nihropenres.13674.2)
Supplement: Peer review history

# Reviewer report: Reviewer response for version 2

**DOI:** 10.3310/nihropenres.15418.r38622

**Published:** 2026-01-06

**Authors:** Elizabeth B. Burgener

**Affiliations:** 1 Pediatric Pulmonology, Children's Hospital Los Angeles, University of Southern California Keck School of Medicine, Los Angeles, California, USA

The authors have made appropriate and responsive edits and additions.  This manuscript presents data which will be very useful for those developing tests and therapeutics for those living with CF!

Is the work clearly and accurately presented and does it cite the current literature?

Partly

If applicable, is the statistical analysis and its interpretation appropriate?

Not applicable

Are all the source data underlying the results available to ensure full reproducibility?

Yes

Is the study design appropriate and is the work technically sound?

Yes

Are the conclusions drawn adequately supported by the results?

Yes

Are sufficient details of methods and analysis provided to allow replication by others?

Yes

Reviewer Expertise:

chronic airway infection, cystic fibrosis, pseudomonas, bacteriophage

I confirm that I have read this submission and believe that I have an appropriate level of expertise to confirm that it is of an acceptable scientific standard.

# Reviewer report: Reviewer response for version 1

**DOI:** 10.3310/nihropenres.14847.r37737

**Published:** 2025-11-10

**Authors:** Morven Brown

**Affiliations:** 1 Newcastle University, Newcastle upon Tyne, UK

This is an interesting and pragmatic study exploring the views of patients/carers and clinicians regarding the diagnosis and management of pulmonary infections in cystic fibrosis.

 There is a relatively small sample, particularly of patients, however, this is a very applied study which has been carried out as part of a larger project (which involved surveys which were informed by this qualitative work) to inform the characteristics that new diagnostic tests should have/are important to patients and clinicians. In the plain summary it mentions that the 'findings were used as part of wider project which included surveys and a symposium event'. However, this detail is not repeated in the main body of the paper. Rather at the conclusion it is just stated that the 'findings were taken forward and addressed in the subsequent stages of the project'. It would be useful to have a little more detail here e.g., that the findings informed the questionnaire and in what way.

 It is stated that analysis was 'performed based on the recordings' which is a little unusual. Was this part of a rapid analysis process?

 'Kaftrio' is mentioned in the quotes. I assuming this an ETI but clarification in the text/quote would be useful.

 There are a few instances where there are abbreviations which need to be clarified e.g., UK NHS in the abstract; CF in the plain language summary; pwCF in the introduction; UK NHS in the introduction.

 There is repetition at the bottom of page 4 and page 5 in the 'Focus Group Format' of the consent forms and information sheets being drafted and revised.

 There are a lot of abbreviations, but the abbreviation list at the end of the paper is very useful.

Is the work clearly and accurately presented and does it cite the current literature?

Yes

If applicable, is the statistical analysis and its interpretation appropriate?

Not applicable

Are all the source data underlying the results available to ensure full reproducibility?

Yes

Is the study design appropriate and is the work technically sound?

Yes

Are the conclusions drawn adequately supported by the results?

Yes

Are sufficient details of methods and analysis provided to allow replication by others?

Yes

Reviewer Expertise:

Qualitative researcher; health psychology

I confirm that I have read this submission and believe that I have an appropriate level of expertise to confirm that it is of an acceptable scientific standard.

# Response

**Published:** 2025-11-26

**Authors:** Nicola Howe

**Affiliations:** Faculty of Medical Sciences, Newcastle University, Newcastle upon Tyne, England, UK

We would like to thank the reviewer for the time taken to review. We have responded to comments and made changes to the manuscript where applicable.

**There is a relatively small sample, particularly of patients, however, this is a very applied study which has been carried out as part of a larger project (which involved surveys which were informed by this qualitative work) to inform the characteristics that new diagnostic tests should have/are important to patients and clinicians. In the plain summary it mentions that the 'findings were used as part of wider project which included surveys and a symposium event'. However, this detail is not repeated in the main body of the paper. Rather at the conclusion it is just stated that the 'findings were taken forward and addressed in the subsequent stages of the project'. It would be useful to have a little more detail here e.g., that the findings informed the questionnaire and in what way.**

 In response to another reviewer, we have now added Figure 1, which highlights the focus groups in the context of the wider TPP development project.

 We are also now in a position to reference our methodological paper which details the way in which the focus groups contributed to subsequent stages of TPP development. We have added some text to make it clearer that these focus groups were part of a wider project in the introduction (page 4, para 3& 4) and discussion (page 33, paragraph 2).

**It is stated that analysis was 'performed based on the recordings' which is a little unusual. Was this part of a rapid analysis process?**

 Thank you for identifying this error. We have reworded the abstract to make it clear that analysis was conducted on the data collected during the focus groups. Analysis was not performed on the recordings. We did refer back to the recordings to clean and validate the transcripts.

**'Kaftrio' is mentioned in the quotes. I assuming this an ETI but clarification in the text/quote would be useful.**

 We have added in brackets after mentions of ‘kaftrio’ that it is indeed an ETI.

**There are a few instances where there are abbreviations which need to be clarified e.g., UK NHS in the abstract; CF in the plain language summary; pwCF in the introduction; UK NHS in the introduction.**

 We have now defined these abbreviations.

**There is repetition at the bottom of page 4 and page 5 in the 'Focus Group Format' of the consent forms and information sheets being drafted and revised.**

 We have now removed this duplication and streamlined the methods section.

**There are a lot of abbreviations, but the abbreviation list at the end of the paper is very useful.**

 Thank you. We have checked that all abbreviations appear in this list.

# Reviewer report: Reviewer response for version 1

**DOI:** 10.3310/nihropenres.14847.r37739

**Published:** 2025-11-08

**Authors:** Elizabeth B. Burgener

**Affiliations:** 1 Pediatric Pulmonology, Children's Hospital Los Angeles, University of Southern California Keck School of Medicine, Los Angeles, California, USA

This manuscript seeks to describe the desired product for diagnosing infection and pulmonary exacerbations in cystic fibrosis (CF).  The authors do so by conducing 3 focus groups that consist of people living with CF (pwCF) and family members and caregivers for pwCF and medical providers who care for pwCF.  The responses collected during these focus groups were summarized with some context setting.  I think the intended audience would be academic researchers and biotech companies looking to make an infection diagnostic (though it is not stated).  

 My comments for an improved manuscript are as follows: 

1. The title is long and confusing. I had to read it multiple times to figure out what the article was about.  It would benefit from a more concise and direct title.  
2. The title states it will develop TPP for diagnosing infection AND exacerbations.  Exacerbations in CF are a clinical diagnosis and not made my detecting an infectious agent.  I personally think diagnosing exacerbations is a totally different topic and out of the scope of this manuscript.  
3. The article is wordy and repetitive with inconsistent organization.  The methods section could be shortened significantly, by eliminating repetition and would then be much easier to read.  
4. The results are organized into sections where some are about specific tests and others about specific desires of the focus groups.  I would recommend grouping comments/desires into themes and then each section would be a general desire and stated as such.  
5. There is statements about standard of care a well as italicized sections describing current tests which are repetitive and inconsistent for each section.  I would recommend eliminating that organization and providing context where necessary for setting the scene for stated desires but remove most of the “factual” sections. 
6. While very important for CF care teams and deliver of care, the sections on team and patient interaction, rapport building and shared decision making are not relevant to this paper’s intention which was to describe at TPP for infection detection.  If the authors really want to include that data that was captured, perhaps a last section on other stated desires or comments on CF care.  Similarly the section on equity and funding structures is not presented in a way that is relevant to TPP of infection diagnostics.  
7. The discussion of MRI is misleading.  Reading this makes it sound like MRI is common in evaluation of pulmonary disease in CF clinical care, it is even mentioned in is done in the emergency room in a section describing MRIs (which is not the case).  This section would benefit from a clinician’s involvement.  
8. Project Breathe is mentioned multiple times.  I would not assume the reader is familiar with that project. This reviewer is not familiar with it.  
9. The section on blood tests is very vague and discusses different types of tests together in a way that doesn’t make sense.  Again this section would benefit from a clinician’s involvement.  
10. None of the authors are clinicians and it shows.  I would recommend involving a pulmonologist to assist in re-writing/organizing this manuscript to give better context to the CF and organize in a way that is easier to read.  
11. I am not trained in qualitative research.  I suggest an additional reviewer with that expertise is recruited to provide a review of the methods.

Is the work clearly and accurately presented and does it cite the current literature?

Partly

If applicable, is the statistical analysis and its interpretation appropriate?

Not applicable

Are all the source data underlying the results available to ensure full reproducibility?

Yes

Is the study design appropriate and is the work technically sound?

Yes

Are the conclusions drawn adequately supported by the results?

Yes

Are sufficient details of methods and analysis provided to allow replication by others?

Yes

Reviewer Expertise:

chronic airway infection, cystic fibrosis, pseudomonas, bacteriophage

I confirm that I have read this submission and believe that I have an appropriate level of expertise to confirm that it is of an acceptable scientific standard, however I have significant reservations, as outlined above.

# Response

**Published:** 2025-11-26

**Authors:** Nicola Howe

**Affiliations:** Faculty of Medical Sciences, Newcastle University, Newcastle upon Tyne, England, UK

We would like to thank the reviewer for the time taken to review. We have responded to comments and made changes to the manuscript where applicable.

**The title is long and confusing. I had to read it multiple times to figure out what the article was about.  It would benefit from a more concise and direct title.**

 We have rearranged and shortened the title to read: “Unmet diagnostic needs in cystic fibrosis infections and exacerbations: Focus groups to inform target product profiles (TPPs)” as we believe this is clearer.

**The title states it will develop TPP for diagnosing infection AND exacerbations.  Exacerbations in CF are a clinical diagnosis and not made my detecting an infectious agent.  I personally think diagnosing exacerbations is a totally different topic and out of the scope of this manuscript. ** 

 This manuscript reports the findings of focus groups that were a component of a wider project to develop target product profiles for infection and for exacerbations in CF. The scope was developed through elicitation exercises with patients and clinical care teams who identified need for diagnostics for both exacerbations and infections. For exacerbation, diagnostics would provide support or to the clinical diagnosis mentioned by the reviewer. We subsequently developed multiple TPPs and these did cover both exacerbation and infection, but separately. We recognised that pathways, and therefore suitable diagnostics, will differ between the two.

**The article is wordy and repetitive with inconsistent organization.  The methods section could be shortened significantly, by eliminating repetition and would then be much easier to read.**

 We have removed some repetition in the methods (focus group format and analysis) and streamlined this section.

**The results are organized into sections where some are about specific tests and others about specific desires of the focus groups.  I would recommend grouping comments/desires into themes and then each section would be a general desire and stated as such. ** 

 We have presented our results as themes and sub themes identified inductively during the analysis. Even those themes that cover discussion of existing tests are still opinions of clinical and pwCF participants. Based on the reviewer recommendations we have removed the contextual ‘factual’ information around these to make this clearer. Some themes cover preferred test characteristics and some encompass comments and desires but we have laid them out theme by theme in the manner in which they were identified.

**There is statements about standard of care a well as italicized sections describing current tests which are repetitive and inconsistent for each section.  I would recommend eliminating that organization and providing context where necessary for setting the scene for stated desires but remove most of the “factual” sections.**

 Each of the current diagnostic testing methods included in the manuscript were those identified by participants. Details on the use, accuracy and acceptability were given by participants themselves. However, we had added the information in italics to contextualise the current placement and use of diagnostics for readers. Based on the reviewer comment we have now moved this contextual information to a reference table (Table 4) and clarified in the results section which data is from participants and which is from the authors (page 7, results, paragraph 1 & page 9, current diagnostic tests, paragraph 2). This also reduces the volume of data under this theme.

**While very important for CF care teams and deliver of care, the sections on team and patient interaction, rapport building and shared decision making are not relevant to this paper’s intention which was to describe at TPP for infection detection.  If the authors really want to include that data that was captured, perhaps a last section on other stated desires or comments on CF care.**

**Similarly the section on equity and funding structures is not presented in a way that is relevant to TPP of infection diagnostics. ** 

 Where topics discussed deviated or drifted slightly from the topic guides, we have still reported these finding for transparency. Our themes were data driven, so the theme, and importance of interactions between patient and provider interactions was drawn from analysis iteratively.

 We believe that this data can be an important consideration for TPP development, moving beyond diagnostic development, towards care pathway and shared decision making and we have now reported this in the discussion (page 36, discussion, paragraph 1). Rapport building can be important in building trust between pwCF and clinicians, especially as low accuracy tests could potentially reduce confidence in healthcare. These themes may also be important for other qualitative researchers, who may wish to repurpose the data and so we have made our transcripts and topic guides publicly available.

 Again, sub- themes on equity and funding were data driven and we reported all our findings for transparency. We also believe that equity and funding structures can also be highly relevant in TPP development, and these factors were included in the resulting TPPs in the sections ‘Equity & ease of access’. However, we do understand the reviewer’s point, and we have therefore clarified in the manuscript (page 35, discussion, paragraph 2) how these factors are of importance and how they influenced TPP development.

**The discussion of MRI is misleading.  Reading this makes it sound like MRI is common in evaluation of pulmonary disease in CF clinical care, it is even mentioned in is done in the emergency room in a section describing MRIs (which is not the case).  This section would benefit from a clinician’s involvement.**

 All of the current methods of diagnostics were identified and described by participants themselves (which we have now made clearer in the methods section).

 We have reviewed the transcripts in relation to discussion of use of MRI and can confirm that we have reported accurately what was stated by participants. However, we have rephrased this section very slightly to make it a bit clearer than we had already that use of MRI for diagnosis is not routine in most NHS trusts and is not directed in current NICE guidelines for management of CF but are performed in some trusts in certain cases.

**Project Breathe is mentioned multiple times.  I would not assume the reader is familiar with that project. This reviewer is not familiar with it.**

 Thank you for identifying this oversight. We have added a reference to the project.

 Project Breathe is an NHS trial which uses artificial intelligence and multiple wearables and digital health technologies such as smartphones or smartwatches to try to predict severe infections and improve lives of people living with chronic lung conditions: https://www.lifearc.org/2023/nhs-trial-uses-artificial-intelligence-to-predict-severe-infections-and-transform-lives-of-people-living-with-chronic-lung-conditions/

**The section on blood tests is very vague and discusses different types of tests together in a way that doesn’t make sense.  Again this section would benefit from a clinician’s involvement.**

 We have reviewed the transcripts in relation to any discussion of blood tests, and can confirm that we have reported accurately what our clinical and pwCF participants said during the focus groups. For both pwCF and clinical experts talk of ‘blood tests’ or ‘blood counts’ was often quite vague usually referring to white cell or full blood counts. Although more blood focussed diagnostics were thought to be a good idea, there was no indication of what exactly these tests might be. Discussion focussed largely on acceptability or placement in the care pathway with few references to specific markers or measures. 

**None of the authors are clinicians and it shows.  I would recommend involving a pulmonologist to assist in re-writing/organizing this manuscript to give better context to the CF and organize in a way that is easier to read.**

 We have added a sentence to the ‘limitations’ section to make clear that the authors are not clinicians, but are qualitative researchers with extensive experience in healthcare research, and that this may have affected our interpretation of the focus group data.  However, our clinical focus groups did include clinical and healthcare staff who are experienced in cystic fibrosis and the work was informed throughout by an external advisory group which included primary and secondary care clinicians (including pulmonologists and CF specialists)  who ensured that there was clinical input into each aspect of the TPP development. 

**I am not trained in qualitative research.  I suggest an additional reviewer with that expertise is recruited to provide a review of the methods.**

 A specialist in qualitative methods has also reviewed the article and we have taken their comments on board.

# Reviewer report: Reviewer response for version 1

**DOI:** 10.3310/nihropenres.14847.r34834

**Published:** 2025-03-25

**Authors:** Freddy Frost

**Affiliations:** 1 Respiratory Department, University of Liverpool, Liverpool, England, UK

Thank you for asking me to review this manuscript. This is a relatively straightforward descriptive study of a TPP process and capture key thematic outputs from the process.

 I think the work is timely and relevant and is reported in a systematic way throughout. I have only a minor suggestions

 Comments:

 The work would benefit from a figure to describe the study process to the reader.

Is the work clearly and accurately presented and does it cite the current literature?

Yes

If applicable, is the statistical analysis and its interpretation appropriate?

Not applicable

Are all the source data underlying the results available to ensure full reproducibility?

Yes

Is the study design appropriate and is the work technically sound?

Yes

Are the conclusions drawn adequately supported by the results?

Yes

Are sufficient details of methods and analysis provided to allow replication by others?

Yes

Reviewer Expertise:

Respiratory infection research,  I am not an expert in qualitative or thematic analyses.

I confirm that I have read this submission and believe that I have an appropriate level of expertise to confirm that it is of an acceptable scientific standard.

# Response

**Published:** 2025-11-26

**Authors:** Nicola Howe

**Affiliations:** Faculty of Medical Sciences, Newcastle University, Newcastle upon Tyne, England, UK

We would like to thank the reviewer for their review and kind description of our manuscript.

 We agree that a figure would be useful for explanatory purposes and as per your comment we have added a figure placing the focus group study in context of the wider works to develop a target product profile (TPP). Please see Figure 1. We have amended the manuscript text to refer to this figure.
